# A Hybrid Computer-aided-diagnosis System for Prediction of Breast Cancer Recurrence (HPBCR) Using Optimized Ensemble Learning

**DOI:** 10.1016/j.csbj.2016.11.004

**Published:** 2016-12-06

**Authors:** Mohammad R. Mohebian, Hamid R. Marateb, Marjan Mansourian, Miguel Angel Mañanas, Fariborz Mokarian

**Affiliations:** a Biomedical Engineering Department, Engineering Faculty, University of Isfahan, Hezar Jerib St., 81746-73441, Isfahan, Iran; b Department of Automatic Control, Biomedical Engineering Research Center, Universitat Politècnica de Catalunya, BarcelonaTech (UPC), C. Pau Gargallo, 5, 08028 Barcelona, Spain; c Department of Biostatistics and Epidemiology, School of Public Health, Isfahan University of Medical Sciences, Hezar Jerib St., 81745 Isfahan, Iran; d Cancer Prevention Research Center, Isfahan University of Medical Sciences, Isfahan, Iran; e Department of Internal Medicine, School of Medicine, Isfahan University of Medical Sciences, Isfahan, Iran

**Keywords:** CAD, computer-aided diagnosis, DT, decision tree, FH, family history of cancer, HPBCR, the proposed hybrid predictor of breast cancer recurrence, HRT, hormone therapy, I. Node, number of involved axillary lymph nodes, NR, lymph node involvement ratio, T. Node, number of dissected axillary lymph nodes, TS, tumor size, XRT, radiotherapy, Breast cancer, Cancer recurrence, Computer-assisted diagnosis, Machine learning, Prognosis

## Abstract

Cancer is a collection of diseases that involves growing abnormal cells with the potential to invade or spread to the body. Breast cancer is the second leading cause of cancer death among women. A method for 5-year breast cancer recurrence prediction is presented in this manuscript. Clinicopathologic characteristics of 579 breast cancer patients (recurrence prevalence of 19.3%) were analyzed and discriminative features were selected using statistical feature selection methods. They were further refined by Particle Swarm Optimization (PSO) as the inputs of the classification system with ensemble learning (Bagged Decision Tree: BDT). The proper combination of selected categorical features and also the weight (importance) of the selected interval-measurement-scale features were identified by the PSO algorithm. The performance of HPBCR (hybrid predictor of breast cancer recurrence) was assessed using the holdout and 4-fold cross-validation. Three other classifiers namely as supported vector machines, DT, and multilayer perceptron neural network were used for comparison. The selected features were diagnosis age, tumor size, lymph node involvement ratio, number of involved axillary lymph nodes, progesterone receptor expression, having hormone therapy and type of surgery. The minimum sensitivity, specificity, precision and accuracy of HPBCR were 77%, 93%, 95% and 85%, respectively in the entire cross-validation folds and the hold-out test fold. HPBCR outperformed the other tested classifiers. It showed excellent agreement with the gold standard (i.e. the oncologist opinion after blood tumor marker and imaging tests, and tissue biopsy). This algorithm is thus a promising online tool for the prediction of breast cancer recurrence.

## Highlights

• Breast cancer is the second leading cause of cancer death among women.
• Recurrence prediction could be used by clinicians to make proper treatment plan.
• A hybrid prognosis tool with optimized ensemble learning is proposed in this paper.
• 579 breast cancer patients (with the recurrence prevalence of 19.3%) were analyzed.
• The average accuracy and precision were 89.2 ± 0.6% and 96.5 ± 1.0%, respectively.

## 1 Introduction

Computer-aided diagnosis (CAD) is using computers and software to interpret medical information. The purpose of CAD is to improve the diagnosis accuracy. In fact, CAD is used as a second opinion by the physicians to make the final diagnosis decision [1], [2].

Nowadays, CAD is used in many different fields in medicine including, but not limited to, early detection of breast cancer [3], lung cancer diagnosis [4], arrhythmia detection [5] and dental and maxillofacial lesions diagnosis [6]. Several studies have been reported in the literature focusing on the application of CAD for cancer diagnosis and prognosis [7], [8].

Cancer is a collection of diseases that involves growing abnormal cells with the potential to spread to other parts of the body [9]. There are over 200 types of cancer. The most common types of cancer in women are breast, colorectal, lung, and cervical [10].

Cancer is the leading cause of death worldwide, accounting for 8.2 million deaths in 2012 [11]. The most common causes of cancer death are: lung (1.59 million deaths), liver (745,000), stomach (723,000), colorectal (694,000), breast (521,000), and esophageal (400,000) [11]. More than 60% of world's total new annual cancer cases occur in Africa, and Central and South America [12]. The cancer incidence in Asia is also high [13]. It is expected that annual cancer cases will rise from 14 million in 2012 to 22 million within the next 2 decades [11], [12]. Worthwhile, among all types of cancer, breast cancer is the second leading cause of cancer death among women [14].

In Iran, cancer is the third cause of death after coronary heart disease, and accidents [15]. Breast cancer is the leading type of cancer in Iranian females, accounting for 24.6% of all cancers. In Iran, the average women age with breast cancer is 49.6 years [16]. Among all of the provinces in Iran, Isfahan Province is the biggest and most important area located at a desert border in the center of Iran [17]. The rate of cancer is increasing rapidly in Isfahan Province. Cancer control and comprehensive prevention plan is thus necessary [18].

Breast cancer, a complex heterogeneous disease, occurs with a set of different clinical symptoms. It is usually diagnosed using blood tests, MRI, mammography, and CT scan and biopsy. The pathology results from biopsy samples indicate whether the suspicious area is cancerous. Cancer patients, then, undergo a systematic treatment procedure dependent on the cancer stage and additional lab tests such as hormone receptor status [19].

Cancer staging (assigning an ordinal numbers I–IV) is in fact the process to determine the extent to which a cancer has been spread and is based on the following four characteristics: 1) the tumor size, 2) whether the cancer is invasive or non-invasive, 3) the spread of the tumor into the lymph nodes, and 4) the spread of the tumor into other parts of the body (i.e. metastasis). Stage I is an isolated cancer (tumor size ≤ 20 mm in greatest dimension) while stage IV is a metastasis cancer. Most cancer deaths are due to cancer that has spread from its primary site to other organs [12], [20].

Breast cancer is usually treated with surgery, which may be followed by chemotherapy, radiation, and hormone therapies [21]. When the cancer patient is initially treated, the disease can recur at any time. However, most recurrences happen in the first 5 years after treatment [22]. The recurrence could be local (near the mastectomy scar), regional (spread to nearby lymph nodes) or metastatic (spread to other parts of the body not near the breast). Some of the most common sites of recurrence outside the breast are the lymph nodes, bones, liver, lungs, and brain [23].

An important issue is whether we can optimize the treatments to increase the therapeutic efficacy. In fact, 5-year recurrence-free survival is an important treatment quality measure. In principle, it is possible to predict 5-year cancer recurrence using clinicopathologic characteristics of cancer patients [24]. Such a prediction could be used by doctors to make proper treatment plan to considerably prolong patient life [25].

The prediction systems were used for cancer diagnosis in the literature [7]. However, there are few studies focusing on cancer prognosis (including recurrence or survival analysis). Since the focus of the current study is prediction of cancer recurrence, the literature review on cancer recurrence prediction models is provided. Meanwhile, the table of available methods was provided in the Supplementary material S1.

Zeng suggested a mixture classification model containing a two-layer structure called mixture of rough set and support vector machine (SVM) for breast cancer prognosis with the average accuracy of 91% [26].

The Nottingham prognostic index (NPI) is a prognostic regression model proposed by Galea et al. [27] based on tumor size, histological grade, and lymph node status. The NPI calculation equation is as follows: tumor size (cm) × 0.2 + histological grade + lymph node point (negative nodes = 1; 1–3 positive nodes = 2; ≥ 4 positive nodes = 3). The patients were then classified into the low-risk (NPI point < 3.4) and high-risk groups (NPI point ≥ 3.4) [27], [28].

Kim et al. [28] used normalized mutual information index for feature selection and supported vector machines (SVM), Cox-proportional hazard regression model, and artificial neural network classifiers for classification in a sample size of 679 patients (the recurrence prevalence of 28.6%). The following features were used in their prognosis system: local invasion of tumor, number of tumors, number of metastatic lymph nodes, the histological grade, tumor size, estrogen receptor, and lymphovascular invasion and reached the sensitivity, specificity and area under the curve of 89%, 73% and 0.85, respectively for the best classifier (SVM). Although the statistical power of their system is acceptable (Power = 89% > 80%), the Type I error is beyond the acceptable range (α = 0.17 > 0.05).

Ahmad et al. [29] used the SVM, decision tree, and multilayer perceptron artificial neural network classifiers with feature selection in a sample size of 547 patients (the recurrence prevalence of 21.4%). The predictors were age at diagnosis, menarche and menopause, tumor size, number of involved and dissected axially lymph nodes, grade and HER2. The best classifier (SVM) had sensitivity, specificity and accuracy of 96%, 91% and 94%, respectively. Despite the high performance of the algorithm proposed by the authors, cases with primary metastasis, a metastasis diagnosed at the time of registry, were not excluded from the analysis. In fact, having metastasis was one of the attributes primarily used by their proposed system. This necessary exclusion has been implemented in similar studies [28], [30]. The reason for such an exclusion is that the survival rate of patients with stage IV (metastasis) breast cancer within 5 years is about 10% to 15%. Thus, most of patients with primary metastasis experience cancer recurrence.

The discriminative prognosis predictors vary regionally in different studies [27], [28], [29]. Also, such prognosis tools have been recently used to optimize the treatment protocol. An example is including the cancer recurrence risk to breast cancer treatment guidelines by the American Society of Clinical Oncology (ASCO) and the National Comprehensive Cancer Network (NCCN). Thus, there is a need to design prognosis tools in regions that are different based on cancer epidemiology. Meanwhile, it is necessary to validate the cancer prognosis system using extensive diagnosis validation criteria. Therefore, the aim of this study was to *reliably* and *accurately* predict breast cancer recurrence using pathological and demographics features of the patients. In the proposed CAD system, HPBCR (hybrid predictor of breast cancer recurrence), a hybrid technique including statistical features selection, meta-heuristic population-based optimization and ensemble learning were used to predict breast cancer recurrence in the first 5 years after the diagnosis.

The rest of the paper is organized as follows: in the next section, information about the experimental protocol and the pattern recognition methods used in this study is presented. Section 3 provides the results of HPBCR and its comparison with the state-of-the-art. The discussion is provided in Section 4 and finally, the conclusions are summarized in Section 5.

## 2 Materials and Methods

### 2.1 The Cohort Dataset

In this study we used a 16-year registry cohort database (1998–2014) on 1085 women who diagnosed with breast cancer in Isfahan Sayed-o-Shohada cancer research center. History of clinical conditions and therapy of patients was continued until death or lost to follow up. Characteristics of variables and tumor have been recorded by interview and reported as pathology results. Time to recurrence was based on the physicians' opinion. The following information was extracted from each patient: age at diagnosis of breast cancer, lymph node involvement ratio (NR) defined as ratio of involved to dissected lymph nodes [31], age of menarche, number of pregnancy (No. Preg), primary tumor size (TS), cellular marker for proliferation (Ki67), number of involved (I. Node) and dissected (T. Node) axillary lymph nodes, number of chemotherapies (No. Chemo) and categorical covariates of family history of cancer (FH: negative/positive), having more than one tumor in the breast (multifocal: negative/positive), estrogen receptor expression (ER: negative (also known as absent)/positive), progesterone receptor expression (PR: negative (also known as absent)/positive), tumor protein 53 (p53: negative/positive), type of surgery (MRM: modified radical mastectomy, BCS: breast-conserving surgery, Mast: mastectomy), epidermal growth factor receptor-2 (Her2: negative/positive (also known as overexpressed)), Cathepsin-D protein status (Cathepsin: negative/positive), hormone therapy (HRT: negative/positive), radiotherapy (XRT: negative/positive) and the histological grade of tumor (1 to 4). Moreover, five main molecular subtypes of breast cancer were extracted as shown to affect recurrence prognosis in the literature. They were calculated using ER, PR, HER2 and Ki-67 (the cutoff value of 14% to define ‘low’ and ‘high’) status. These subtypes are: luminal A (ER and/or PR-positive/HER2 negative/low Ki-67), luminal B (ER- and/or PR-positive/HER2-negative/high Ki-67), HER2-positive luminal B (ER- and/or PR-positive/HER2 positive/any Ki-67), non-luminal HER2-positive (ER and PR negative/HER2 positive) and triple negative (ER and PR negative/HER2-negative) [32]. The brief description of the above features used in our study was provided in the Supplementary material S2.

Eighty nine subjects were excluded from the analysis because of primary metastasis, a metastasis diagnosed at the time of registry [30]. A number of 579 breast cancer patients with complete information were involved in our analysis. All subjects gave informed consent to the experimental procedure. The experimental protocol was approved by the Isfahan University of Medical Sciences Panel on Medical Human Subjects and conformed to the Declaration of Helsinki.

**Fig. 1:**
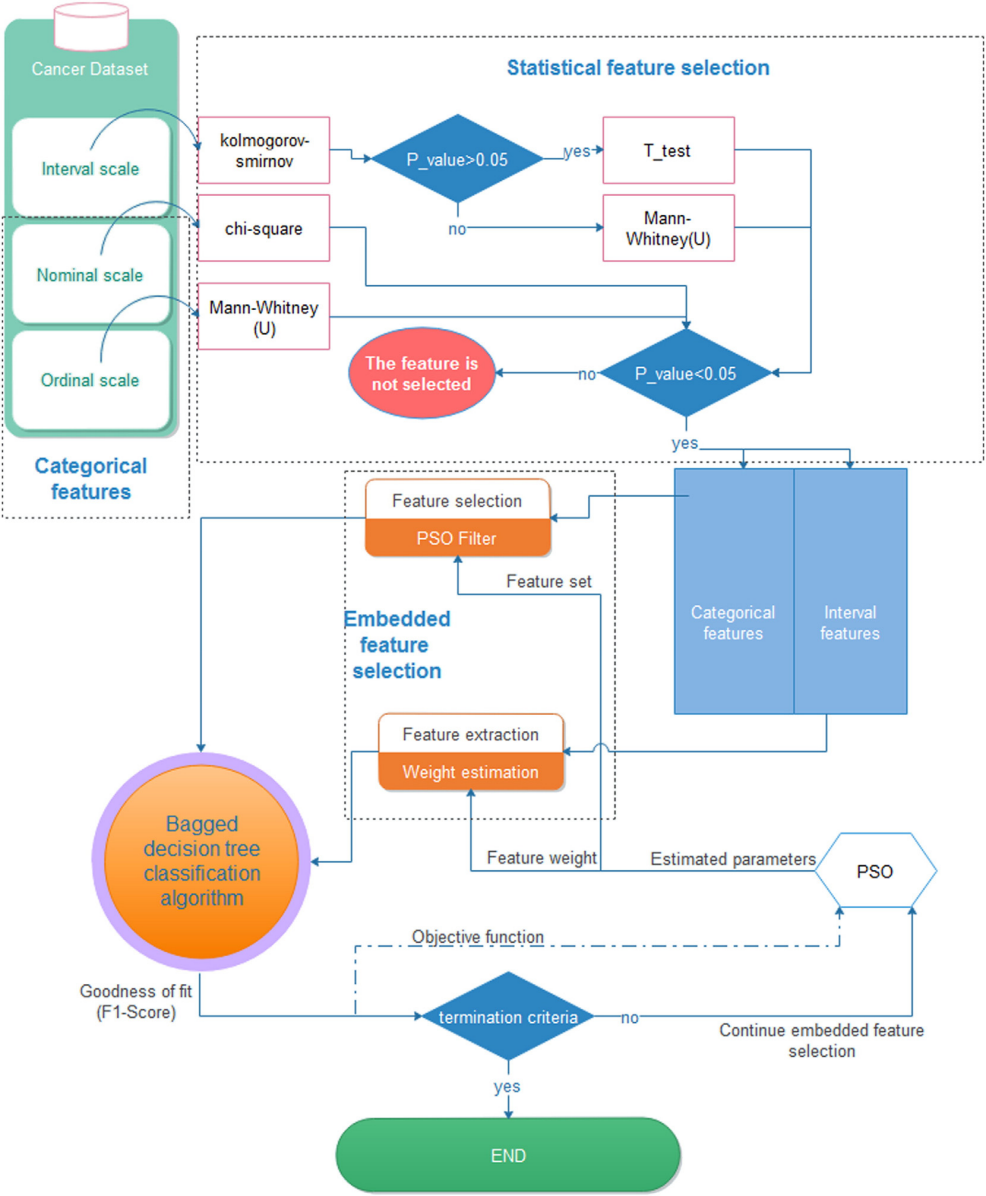
The structure of the proposed prognosis system (HPBCR). Other classifiers such as SVM and MLP could be used instead of BDT. The pseudo-code of HPBCR is provided in the Supplementary material S3. The input features in HPBCR were: diagnosis age, nodal ratio, menarche age, the number of pregnancy, tumor size, Ki67, the number of involved and dissected nodes, as interval features, type of surgery, molecular subtypes, family history of cancer, multifocal tumor, estrogen and progesterone receptor status, p53 mutation, Her2 expression, Cathepsin-D protein status, using hormone therapy, and radiotherapy as nominal features and the tumor grade as an ordinal feature. Briefly, the input features are first selected using statistical feature selection. The selected features were used by Bagged Decision Tree to build the classifier. The optimal feature set and the weight of features are estimated using Particle Swarm Optimization during learning. The algorithm stops if no significant improvement is seen in the objective function or the maximum number of iterations (set to 100 in our study) is reached. The structure of the proposed prognosis system (HPBCR). Other classifiers such as SVM and MLP could be used instead of BDT. The pseudo-code of HPBCR is provided in the Supplementary material S3. The input features in HPBCR were: diagnosis age, nodal ratio, menarche age, the number of pregnancy, tumor size, Ki67, the number of involved and dissected nodes, as interval features, type of surgery, molecular subtypes, family history of cancer, multifocal tumor, estrogen and progesterone receptor status, p53 mutation, Her2 expression, Cathepsin-D protein status, using hormone therapy, and radiotherapy as nominal features and the tumor grade as an ordinal feature. Briefly, the input features are first selected using statistical feature selection. The selected features were used by Bagged Decision Tree to build the classifier. The optimal feature set and the weight of features are estimated using Particle Swarm Optimization during learning. The algorithm stops if no significant improvement is seen in the objective function or the maximum number of iterations (set to 100 in our study) is reached.

The structure of HPBCR is depicted in Fig. 1. First, the statistical features' selection was used to identify significant discriminative features. Among such features, categorical features (i.e. nominal or ordinal measurement scales [33]) used in the Bagged Decision Tree (BGD) were selected by Particle Swarm Optimization (PSO). The features with the interval measurement scale used by BGD were assigned weights being also tuned by PSO. The fitness function of the PSO was the F-score of the classifier on the training set (Eq. (1)). In the following, the details HPBCR are discussed. The pseudo-code of the HPBCR was presented in the Supplementary material S3.

(1) $$\mathrm{fitness}={\mathrm{FScore}}_{\mathrm{train}}$$

### 2.2 Statistical Feature Selection

Feature selection (FS), the process of selecting a subset of relevant features, is divided into wrapper, embedded and filter methods. Filter method selects subsets of variables as a pre-processing step, independently from the chosen classifier while the other two methods, are used during machine learning procedure [34]. In wrapper methods, the selection of a features set is formulated as a search problem, in which different feature combinations are evaluated. Embedded methods, on the other hand, learn which set best contribute to the model accuracy while the model is being created. The former method searches the entire feature subsets while in the later one, search is guided by learning process which is prone to less over-fitting [35].

In fact, Filter FS also known as Statistical FS (SFS) applies a statistical measure to assign a score to each feature [36]. Such features are then ranked and selected based on the resulting score. Univariate SFS was first used in our analysis. Accordingly, the normality of the features with interval measurement scale was assessed using the Kolmogorov–Smirnov (KS) test [37]. Then, independent-sample t-test was used to identify statistically discriminative normally-distributed features. Meanwhile, Wilcoxon Mann–Whitney test was used to distinguish discriminative features with ordinal or non-normal interval measurement scales [38] (Fig. 1).

Chi-square test was used to check whether a nominal-measurement-scale feature was discriminative. The discriminative features were selected and further refined as the inputs of the classification system (BDT), next discussed.

### 2.3 Bagged Decision Tree

Decision tree builds classification models in the form of a tree structure. It divides a dataset into small subsets to incrementally develop an associated decision tree with decision and leaf nodes [39]. It uses entropy to calculate the homogeneity of samples to build the tree [40]. We used the statistical classifier C4.5 decision tree [41] with pruning in HPBCR. C4.5 sorts the data at every node of the tree to determine the best splitting attribute. When the tree is constructed, pruning is carried out from the leaves to the root. The redundant sub-trees are removed and thus the prediction error decreases. C4.5 handles both continuous and discrete attributes and is thus suitable for our application. Moreover, the online implementation of the resulting tree is simple.

Decision tree has been applied to many fields; for example in prediction of breast cancer relapse after surgery [42], classification mass spectrometry of blood serum samples from people with and without lung cancer [43] and gene expression data for cancer classification [44]. In fact, decision trees are reliable and effective decision making techniques used in different areas of medical decision making [45]. Meanwhile, in evidence-based medicine, it was used for clinical decision analysis [46].

Bagging is a method for improving results of machine learning classification algorithms. This method was formulated by Breiman and its name was deduced from the phrase “bootstrap aggregating”. The details of bagging could be found in [47], [48]. Bagging is usually applied to decision tree methods, though it can be used with other classifiers. In summary bagged tree, resamples the training dataset several times (bootstrapping), and builds a decision tree model from each; then aggregate these models together for a final classifier. This method was used for breast cancer classification [49], gene expression data analysis [50] and prostate cancer classification [51]. In our study, the number of trees was set to 10 for bagging.

The proper combination of categorical selected features and also the weight (importance) of the selected interval-measurement-scale features were identified using a meta-heuristics population-based stochastic optimization method entitled as Particle Swarm Optimization (PSO) (Fig. 1), discussed in the next chapter.

### 2.4 Particle Swarm Optimization (PSO)

PSO is an evolutionary computational method inspired by flocking birds [52], applied in many different areas, including manufacturing [53], civil engineering [54], optimum design [55], computational neuroscience [56] and breast cancer detection [57]. PSO is initialized with a population of random solutions known as particles, growing over generations to find optimal solutions. In a population, each particle has a velocity (i.e. rate of change in solutions) to enable them to fly through the problem space. Therefore, each particle is represented by a position and a velocity [58]. The current velocity of a particle depends on its previous velocity and on the distances of the particle from the personal (cognitive term) and neighborhood (social term) best positions (Eq. (2)).

(2) $$v_{i}^{n+1}={\mathrm{wv}}_{i}^{n}+c_{1}r_{1}^{n}\left(p_{i}^{n}-x_{i}^{n}\right)+c_{2}r_{2}^{n}\left(p_{g}^{n}-x_{i}^{n}\right)$$

where *w* is the inertia weight; *c*_1_, *c*_2_ are the cognitive and social acceleration coefficients; *i* is the particle number, *r*_1_ and *r*_2_ are independent random numbers whose elements are uniformly distributed in [0, 1]; *n* denotes the iteration number, *p*_*i*_ is the personal best of the *i*th particle and *p*_*g*_ is the neighborhood best.

Each particle records its best position (personal best) so far and the best position achieved in the group (neighborhood best) among all personal bests. Then, the new position of a particle is determined based on its previous position information and its current velocity (Eq. (3)).

(3) $$x_{i}^{n+1}=x_{i}^{n}+v_{i}^{n+1}$$

The objective is to find the particle that best maximizes the target fitness function (Eq. (1)). Moreover, the neighborhood of each particle is the entire swarm in our method (star topology).

In HPBCR, the acceleration coefficients *c*_1_ and *c*_2_ were set to 2. A large value of inertia weight supports global search (“exploration”), while a small value favors local search (“exploitation”). A suitable strategy is to first motivate exploration, and then exploitation [56]. Thus, the inertia coefficient was set to 1.00 at the first iteration and was linearly decreased with the damping coefficient of 0.99 at each iteration, and the number of particles was set to 20 and 100 iterations were performed. Moreover, in order to increase the convergence possibility, velocity clamping (V_max_ = 4) was used.

PSO algorithm was used to filter categorical features and to estimate the weights of interval features (Fig. 1). In categorical features, the position of the particles was rounded to 0 and 1 (Binary PSO) but for interval features, such values were real numbers between zero and one. The pseudo-code of the embedded feature selection based on PSO was provided in the Supplementary material S3 (section B).

### 2.5 State-of-the-art

In our study, other classification methods namely as MLP, SVM and DT were used for comparison. Such methods were proposed in similar studies in the literature [28], [29]. The selected features using SFS were used as the input to these classifiers.

MLP is a feed-forward artificial neural network model that maps sets of input data onto a set of appropriate outputs. It consists of multiple fully connected layers of nodes in which learning happens by changing connection weights through back propagation. It is a modification of the standard linear perceptron and can be used for classification of data that are not linearly separable [59]. In this study the MLP with 1 hidden layer with 10 neurons and sigmoid activation function was used. Such an activation function was reported by Isa et al. to be suitable for the prediction of breast cancer [60]. Meanwhile, the sensitivity analysis was performed on the number of neurons. Cross-validation on the training set (70% of data) was performed. When increasing the number of neurons up to 10, the prediction accuracy increased and did not improve considerably afterwards. Thus, ten neurons were used as to avoid over-fitting problem.

SVM constructs a hyper plane or set of hyper planes in a high or infinite-dimensional space, which can be used for classification [61]. The SVM algorithm has been widely applied in the biological and many other fields [62], [63], [64]. In our study, the radial basis function (RBF) kernels were used. The soft-margin parameter and the radius of the RBF kernel should be set properly, because inappropriate parameter settings result in poor classification. The method proposed by Wu and Wang [65] was used to set the soft-margin and RBF kernel parameters.

### 2.6 Validation

#### 2.6.1 The Performance Indices for Each Classifier

The performance of the classifiers was assessed using the holdout method, an approach to out-of-sample evaluation, where the dataset was split into two mutually exclusive sets (70% training and 30% test sets). The classifiers were then trained on the first set and tested on the other set [66]. Additionally, 4-fold cross-validation was used to assess the performance of the best classifier to overcome a possible biased error estimate [67]. A comprehensive list of performance indices [68], [69], [70], [71], [72] was selected and reported for each classifier to identify different statistical errors which is crucial in medical diagnosis especially in cancer studies. The performance measures of the classifiers are listed in Table 1, along with their definitions. Discriminant power (DP) was characterized as “poor” when DP < 1, “limited” when DP < 2, “fair” when DP < 3 and “good” in other cases [72], [73]. Among those measures AUC ROC is a global measure of diagnosis accuracy. ROC (receiver operating characteristic) curve is a plot of true positive rate (i.e. sensitivity) versus false positive rate (i.e. 1-specificity) of the diagnosis system as its discrimination threshold is varied. AUC (area under roc curve) is an effective and combined measure of the validity of the diagnosis test, which is also known as balanced accuracy defined the average of the sensitivity and specificity of the diagnosis system [72]. Its ranges for excellent, very good and good diagnosis accuracy are (0.9–1.0), (0.8–0.9) and (0.7–0.8) respectively [74].

Along with such indices, the agreement rate between the output of the classifier and the gold standard was assessed using Cohen's kappa coefficient [75]. Kappas over 0.75 was characterized as excellent, 0.40 to 0.75 as fair to good, and below 0.40 as poor [76]. A diagnosis system was considered as reliable if (1) its sensitivity and specificity were at least 80% (the minimum statistical power of 80%) and 95% (the maximum Type I error of 0.05), respectively [77], [78], and (2) its false discovery rate (FDR = 1-precision) is as low as 5% [79]. Moreover, as a general rule, diagnosis tests with positive likelihood ratio (LR^+^) greater than 10 or negative likelihood ratio (LR^−^) less than 0.1 are preferred [80]. The application of sensitivity and specificity is highly dependent on the prevalence of the disease. A conservative method could be fulfilling both LR conditions (i.e. DOR greater than 100). An illustration of the validation criteria was presented in the Supplementary material S4.

#### 2.6.2 Comparison Between Different Classifiers

McNemar's test, also known as the Gillick test, was used to compare different classifiers to identify whether one classifier statistically significantly outperforms the other or not [67], [81]. It was originally used to analyze retrospective case–control studies, where each case is matched to a particular control or it can be used to analyze experimental studies, where two treatments are given to match subjects [82].

To compare two classifiers A and B, *z* statistics is estimated as:

(4) $$z=\frac{\left|x-y\right|-1}{\sqrt{x+y}}$$

where *x* is the number of samples misclassified by A but not B and *y* is number of samples misclassified by B but not A. The null hypothesis (that the classifiers have the same error) can be rejected (with probability of incorrect rejection of 0.05) if |* z* | > 1.96 [67], [83]. In such a case, a classifier with higher DP value was reported as superior to the other one.

All data processing was performed off-line using Matlab version 8.6 (The MathWorks Inc., Natick, MA, USA). All statistical analysis and calculations were performed using the SPSS statistical package, version 18.0 (SPSS Inc., Chicago, IL, USA).

### 2.7 Online Web-based HPBCR Implementation

Ten extracted rules were obtained from BDT whose feature weights were optimized using PSO. The prognosis system was written in C# language and used in ASP.net5 which is an open-source framework from Microsoft Corporation. The prediction model is freely available in the website (http://www.Prognosis.ir). A snapshot of the developed online program is shown in the Supplementary material S5.

## 3 Results

The average age of the participants was 46.8 ± 10.0 years. Among the number of 579 patients participated in our study, 19.3% had cancer recurrence during five years after diagnosis. The demographic and pathological characteristics of the participants, grouped by their classification with/without recurrence, are depicted in Table 2, Table 3, Table 4 for the features with interval, nominal and ordinal measurement scales, respectively. In fact for the features with binary, nominal with more than two categories, ordinal and interval measurement scales, their existence, category, rank and value is important in the diagnosis system. Among the interval measurement scale features, only age at diagnosis and TS were normally distributed in the recurrence group. Thus, due to the rejection of class dependence normality, Mann–Whitney test was used for SFS in interval features. Selected discriminative features using SFS were: age at diagnosis, NR, TS, I. Node, T. Node, Ki67, PR, HRT, surgery and cancer subtypes.

In our study, discretization was used on the following attribute intervals: age at diagnosis, NR and TS. In machine learning, discretization refers to the procedure of partitioning continuous attributes to discretized variables as to improve the classification performance [84], [85]. We used a set of cut points taken from the literature, indicating the diagnosis properties of individual attributes as below to create categorical variables.

Age was categorized (Age.cat) as less than 40 years and more than 40 years [86]. NR was categorized (NR.cat) in ≤ 0.25 and > 0.25 [87], [88]. TS was also categorized (TS.cat) as ≤ 2 (T1), (2,5) (T2) and > 5 (cm) (T3) [89], [90], [91].

**Fig. 2:**
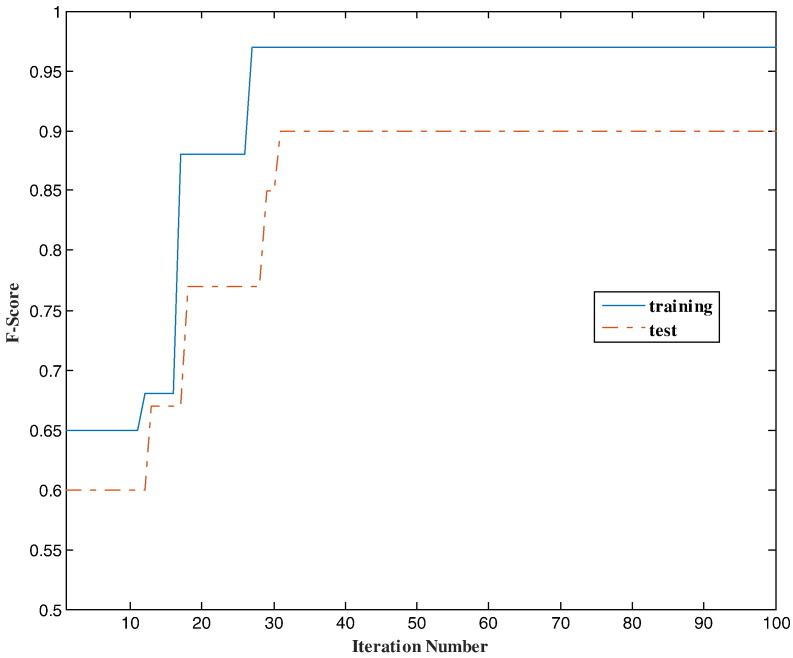
The value of the fitness function (F-Score of the proposed classifier (HPBCR) on the training set-solid line) and the F-Score on the test set (dash-dot line) during optimization procedure. The termination criterion was only the maximum number of iterations (i.e. 100) in this plot.

The value of the fitness function (F-score on the training set) and the test set performance (F-score on the test set) at some iteration were shown in Fig. 2. The following categorical features were kept after running PSO and BDT: PR, HRT, Surgery, age.cat, TS.cat and NR.cat. Among the features with interval measurement scale, Ki67 and T. Node were not selected by BDT. In fact BDT uses entropy principle to choose the best discriminative features [92]. The estimated weight of the selected interval-scale feature I. Node was 0.74.

The performance of the classifiers on the test set (when trained on the training set) using the hold-out validation method was shown in Table 5. The performance of the classifiers was further assessed using 4-fold cross validation (Table 6). The confusion matrix of HPBCR on the test set was shown in Table 7. The performance of HPBCR was statistically significantly higher than that of three other classifiers (McNemar's test; P < 0.05). HPBCR showed limited discriminant power (DP = 1.28), very good diagnosis accuracy (AUC = 0.90), and excellent agreement with the gold standard (Kappa = 0.83). The minimum statistical power and maximum Type I error (α) were 77% and 0.07, respectively in HPBCR (Table 6). Overall, the average statistical power and Type I error of HPBCR was 80% and 0.04, respectively (Table 6). The training time of HPBCR, SVM, DT and MLP was 412.10 ± 14.50 (s), 66.20 ± 11.75 (s), 52.00 ± 4.50 (s) and 132.45 ± 15.50 (s), while the validation time was 0.62 ± 0.31 (s), 0.49 ± 0.24 (s), 0.39 ± 0.33 (s) and 0.49 ± 0.31 (s), respectively. The average running time was the average of 10 runs over 405 and 174 subjects in the training and test sets respectively (hold-out 70%) on an Intel Core-i7 2 GHz CPU with 4 GB of RAM.

## 4 Discussion

### 4.1 The Risk Factors of the Cancer Recurrence

In our study, the following risk factors were selected by HPBCR to predict breast cancer recurrence: Age at diagnosis, TS, NR, I. Node, PR, HRT and type of surgery. Approximately 7% of women with breast cancer are diagnosed before the age of 40 years. It has been shown in the literature that younger age is not only an independent predictor of adverse outcome in survival rate [86], but it is also a risk factor in recurrence metastasis [89], [93]. Bock et al. showed a hazard ratio of 2.8 for local recurrence in patients less than 35 years compared to those above 50 years [94]. Bharat et al. estimated the risk of breast cancer recurrence for women diagnosed below the age of 40 to be 1.53 times higher than in those diagnosed above 40 years [95]. In our study, the first and second age quartile for patients without recurrence was 41 and 47 years compared with 38 and 43 years for those with recurrence. Meanwhile, patients who were less than 40 years old had 33.5% higher hazard of recurrent metastasis in comparison with more than 40 year old patients [89]. In fact, the odds of recurrence in the young (< 40 years) and old (> 40 years) population in our study was 0.43 and 0.19, respectively. Such a trend is in agreement with the literature. Meanwhile, in recurrent cancer patients, the odds ratio (OR) associated with age ≤ 40 compared with age > 40 was 1.8, indicating that discretized age was a good feature for the developed prognosis system.

TS is an independent prognostic factor, with distant recurrence rates increasing with larger tumor size [96]. It has been in fact an important recurrence prognosis factor in several studies [97], [98], [99], [100]. It has been shown in the literature that the median time to the development of metastatic disease shortens as tumor size increases [101], [102], [103]. In our study, the recurrence odds for TS ≤ 2 (T1), 2 < TS ≤ 5 (T2), and TS > 5 (T3) was 0.13, 0.27 and 0.29, respectively showing that increased tumor size is associated with higher probability of cancer recurrence.

NR was shown to be a prognosis factor for cancer recurrence in the literature [100]. Voordeckers et al. showed that NR is one of the main prognosis risk factors for cancer recurrence [104]. Elkhodary et al. showed that the hazard ratio of breast cancer recurrence risk increases when NR increases [105]. Tazhibi et al. indicated that NR (≤ 0.25 vs. > 0.25) is associated with hazard of distance recurrence [89]. In our study, the recurrence odds for the NR ≤ 0.25, and NR > 0.25 was 0.14, and 0.43, respectively showing that increased NR is associated with higher probability of cancer recurrence. Meanwhile, in recurrent cancer patients, the OR associated with NR > 0.25 compared with NR ≤ 0.25 was 1.6, indicating that discretized NR was a good feature for the developed prognosis system.

It was shown in the literature that I. Node is an important predictive factor for recurrence risk [106], [107]. T. Node was also considered a predictor for cancer recurrence [108]. Although SFS selected this feature, it was not used in BDT. One explanation could be that in our dataset, I. Node and T. Node were moderately correlated (r = 0.56; P < 0.001). Also, NR defined as the ratio between I. Node to T. Node, was also used by the classifier. Thus, adding T. Node could not increase the amount of information in the prognosis system. In fact, reducing the number of correlated features improves the classification accuracy [109]. Higher Ki67 expression, on the other hand, was shown to be a prognosis indicator of increased cancer recurrence in the literature [110]. In our study, the second quartile of Ki67 was 14.3 and 17.4 in non-recurrent and recurrent cancer patient, respectably. Although SFS selected this feature, it was not used in BDT either. In our study, Higher Ki67 index tumors showed larger size (TS), and more lymph node involvement (I. Node), which is in agreement with what was reported in the literature [111]. This could be the justification that the final prognosis system did not use Ki67 expression.

PR expression was shown to be an independent recurrence prognostic variable in breast cancer in the literature [112], [113], [114]. Purdie et al. showed that absent PR expression was significantly associated with poor cancer recurrence prognosis [112]. In our study, the recurrence odds for the PR + and PR − was 0.20, and 0.31, respectively which is in agreement with what was mentioned in the literature. Meanwhile, in non-recurrent cancer patients, such odds associated with PR + compared with PR − was 1.6, indicating that PR was a distinctive feature for the developed prognosis system.

It was shown that HRT reduces the risk of breast cancer recurrence in the literature [115]. In our study, the recurrence odds for the HRT + and HRT − was 0.20, and 0.35, respectively which is in agreement with what was mentioned in the literature. Meanwhile, in non-recurrent cancer patients, the recurrence odds associated with HRT + compared with HRT − was 2.4, indicating that HRT was a suitable predictor for the developed prognosis system. It was proposed in the literature that the impact of type of surgery on the breast cancer recurrence might be dependent on age [116]. In our study, the non-recurrence odds for young and old patients underwent MRM surgery was 2.8 and 5.4, respectively. Such odds for the BCS surgery was 4.8 and 8.3. This could in principal support that the performance of the surgery is age dependent.

### 4.2 The Properties and Performance of HPBCR

In our study, among 1085 breast cancer patients, only 579 subjects with complete information and without primary metastasis were included in the prediction. Thus, subjects with missing data were excluded from the analysis. Missing data can introduce bias and reduce efficiency [117]. It is possible to use multiple imputation to handle such incomplete data. Although multiple imputation has potential to improve the validity of medical research, it requires the user to model the missing variable distribution. The validity of the imputed results highly depends on such modeling and underlying assumptions [118].

In our algorithm, SFS was first used to identify the discriminative features. Such a selection was then refined by PSO and also the importance of the interval features, represented by the estimated weights, was identified. Overall, the embedded FS was used in our study where FS is performed as part of the learning procedure. PSO is a meta-heuristics population-based stochastic optimization method. In principle, it is possible to use any other method in this category that could deal with continuous (feature weights) and discreet (feature selection) problems. We also tested genetic algorithm (GA) [119], [120]. In multiple runs, both PSO and GA converged to an identical optimum solution. However, GA required about 150 iterations in comparison with 50 iterations in PSO (Fig. 2), for converging.

An alternative FS method is using multivariate FS namely as multiple logistic regression (MLR), taking into account the relationship between different features. MLR, known as feature vector machine in machine learning, can be used to select statistically significant features [84], [121]. MLR was also tested in our study. However, due to the lack of fit of the MLR model, univariate SFS was used.

In HPBCR, F-score was used as the fitness function. When the dataset is imbalanced, for example in our case where the recurrence prevalence was 19.3, overall accuracy is not a suitable fitness measure. In fact, choosing the wrong objective function while developing a classification model can introduce bias towards majority [122]. Meanwhile HPBCR could be used for other mixed data where each instance in a data set is described by more than one type of measurement scale attribute [33]. In this case, different attributes must be treated differently. In our algorithm, different attributes were manipulated distinctly.

The average sensitivity and specificity of HPBCR are estimated as 80% and 96% (Table 6). Also, considering the maximum Types I (α = 0.07) and II errors (β = 0.23), maximum false discovery rate (FDR = 5%), guarding against testing hypotheses suggested by the data (Type III errors [123]) done by cross-validation, very good diagnosis accuracy (AUC = 0.90), and finally the “excellent agreement” between the gold standard and the outcome of the system (Kappa = 0.83), HPBCR is promising for clinical prognosis tests. HPBCR significantly outperformed the other systems namely as MLP, DT and SVM (McNemar's test; P < 0.05). The other three tested classifiers showed poor DP, good diagnosis accuracy and good agreement with the gold standard (Table 5). The Types I and II errors of the proposed algorithm were better than those obtained by the other classifies (Table 5, Table 6). In HPBCR the average Type I error was less than 0.05 while the average statistical power was higher than 80% in the hold-out (Table 5) and cross validation (Table 6). However, the maximum Type I error (0.07) and minimum power (77%) in the folds used for cross validation were beyond the acceptable clinical ranges and must be improved. The variation of the performance indices in different folds was low (Table 6), showing consistency in the results of HPBCR.

The performance of HPBCR was further compared with the state-of-the-art. Kim et al. [28] used hold-out validation method (70%: training set; 30% test set) and reported the performance of the algorithm on the test set as the AUC of 0.85, sensitivity of 89%, specificity of 73%, precision of 75% (FDR = 25%) and accuracy of 85%. In a similar setting (Table 5), HPBCR had AUC of 0.90, sensitivity of 81%, specificity of 98%, precision of 97% (FDR = 3%) and accuracy of 90%. Our algorithm outperformed the other prediction method in terms of AUC, FDR and accuracy.

NPI method proposed by Galea et al. [27] was implemented and tested in our dataset. The value of the performance measures AUC, sensitivity, specificity, precision, and accuracy were 0.51, 100%, 2%, 25% and 26%, respectively. This method is not suitable in our study because of high Type I error (α = 0.98).

### 4.3 Additional Analysis

The contribution of BDT and PSO to the overall classification performance was also considered. PSO was excluded from the algorithm flowchart and the resulting hold-out performance was reported in Table 8 (Scenario 1), compared with what obtained in the HPBCR (baseline). HPBCR significantly outperformed the modified prognosis system (McNemar's test; P < 0.05). Particularly, discriminant power is deteriorated when excluding PSO in the developed algorithm. Moreover, the modified system is not clinically reliable (Power = 78%, Type I error = 0.09). PSO is in fact used for embedded feature selection and is a necessary section of the program.

Moreover, SVM (Scenario 2) and MLP (Scenario 3) classifiers were used instead of BDT whose features were tuned using PSO. The performance of these two modified prognosis systems was comparable to what obtained in HPBCR (Table 8; McNemar's test; P > 0.05). Thus, BDT could be substituted by SVM and MLP. However, it was shown in the literature that BDT is as good as SVM and MLP [124]. Meanwhile, bagging avoids over-fitting. Using BDT, it was possible to implement HPBCR online efficiently (Section 2.7).

However, the performance of SVM or MLP when PSO is used for embedded feature selection (Table 8) was significantly better than that of SVM or MLP without using PSO (Table 5). This is the reason that hybrid feature selection performed by PSO is essential in the proposed prognosis system. Also, the training time of SVM + PSO, and MLP + PSO was 415.77 ± 10.50 (s) and 710.02 ± 22.55 (s), while the validation time was 0.50 ± 0.27, and 0.50 ± 0.33, respectively (hold-out 70%). Since at each iteration, feature sets are calculated for each 20 particle, the training time is more than cases where PSO is not used.

### 4.4 Final Considerations

This study was a validation of the prognostic performance of HPBCR. The limitation of the current study is that it was a retrospective, single-institution study. The results might have been biased on the basis of available specimen and patterns of referral to the hospital. The sample size must be increased as to improve the statistical power in our prognosis system [125]. The output of the classification system is not fuzzy. It will be useful to add risk assessment to the algorithm and report the risk of recurrence which is the focus of our future activity.

## 5 Conclusions

We proposed a computer-aided prognosis system to identify breast cancer recurrence within 5 years after diagnosis. This method is accurate and precise and could be possibly used by clinicians to make proper treatment plan.

The following are the supplementary data related to this article.

Supplementary material S1 Publications relevant to cancer recurrence prediction. Supplementary material S1

Supplementary material S2 The input features (risk factor) used in the proposed breast cancer recurrence prediction system along with their definition and references. Supplementary material S2

Supplementary material S3 The pseudo code of the proposed algorithm. Supplementary material S3

Supplementary material S4 Statistical analysis of the validation measures. Supplementary material S4

**Supplementary material S5:**
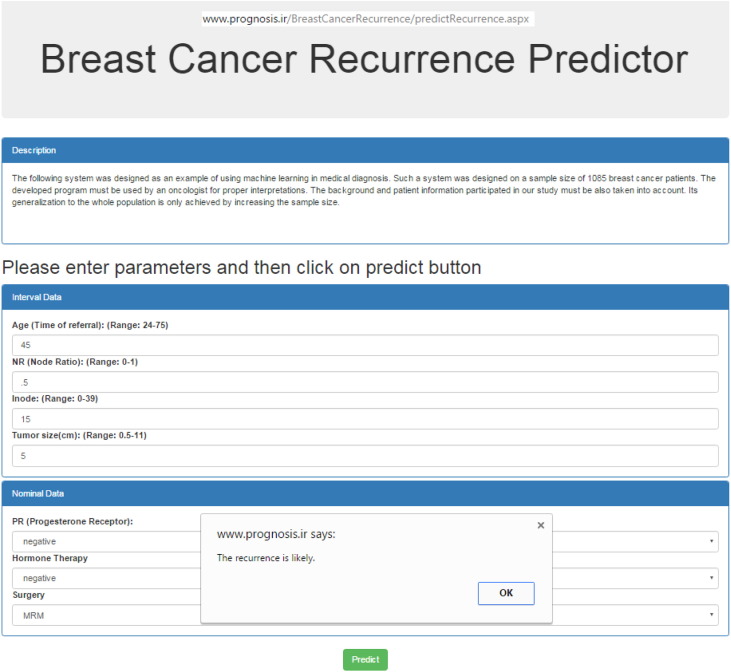
The snapshot of the developed on-line HPBCR. The information of a subject with breast cancer was entered. Based on the prediction, it is likely to have recurrence within 5 years after diagnosis.

## Figures and Tables

**Table 1:** The classification performance measures used in our study.

Se = $\frac{\mathrm{TP}}{\mathrm{TP}+\mathrm{FN}}$	Sp = $\frac{\mathrm{TN}}{\mathrm{TN}+\mathrm{FP}}$	Acc = $\frac{\mathrm{TP}+\mathrm{TN}}{\mathrm{TP}+\mathrm{TN}+\mathrm{FP}+\mathrm{FN}}$
Pr = $\frac{\mathrm{TP}}{\mathrm{TP}+\mathrm{FP}}$	Alpha = 1 − Sp	Beta = 1 − Se
F-score = $\frac{2\times \mathrm{Pr}\times \mathrm{Se}}{\mathrm{Pr}+\mathrm{Se}}$	AUC = $\frac{1}{2}\times \left(\mathrm{Sp}+\mathrm{Se}\right)$	$\mathrm{LR}+=\frac{\mathrm{Se}}{1-\mathrm{Sp}}$
$\mathrm{LR}-=\frac{1-\mathrm{Se}}{\mathrm{Sp}}$	$\mathrm{DOR}=\frac{\mathrm{LR}+}{\mathrm{LR}-}$	
MCC = $\frac{\mathrm{TP}\times \mathrm{TN}-\mathrm{FP}\times \mathrm{FN}}{\sqrt{\left(\mathrm{TP}+\mathrm{FP}\right)\times \left(\mathrm{TP}+\mathrm{FN}\right)\times \left(\mathrm{TN}+\mathrm{FP}\right)\times \left(\mathrm{TN}+\mathrm{FN}\right)}}$		
DP = $\left(\frac{\sqrt{3}}{\pi }\right)\times \log\left(\mathrm{DOR}\right)$		

True positive (TP): subjects with cancer recurrence, correctly identified; false positive (FP): subjects without recurrence, incorrectly identified; true negative (TN): subjects without recurrence, correctly identified; false negative (FN): subjects with recurrence, incorrectly identified; Se: sensitivity = Power; Sp: specificity; Acc: Accuracy; Pr: precision; AUC: area under the receiver operating characteristic (ROC) curve; LR: likelihood ratio; DOR: diagnosis odds ratio; MCC: Matthews correlation coefficient; DP: discriminant power.

**Table 2:** Comparison of clinical and biochemical features (with interval measurement scale) of included subjects with/without cancer recurrence in μ ± σ [min, max].

Variable	With recurrence	Without recurrence
Age⁎ (year)	45.4 ± 10.1 (ND) [27.0, 68.0]	47.2 ± 9.9 [24, 75.0]
NR⁎	0.47 ± 0.36 [0.0, 1.0]	0.24 ± 0.32 [0.0, 1.0]
Menarche	13.3 ± 1.3 [11.0, 17.0]	13.4 ± 1.4 [10.0, 17.0]
No. Preg	3.5 ± 1.9 [0.0, 10.0]	3.7 ± 2.1 [0.0, 10.0]
TS⁎ (cm)	4.0 ± 1.7 (ND) [1.0, 8.5]	3.7 ± 1.8 [0.5, 11.0]
Ki67⁎	21.1 ± 12.6 [6.0, 74.0]	18.0 ± 11.8 [1.0, 86.0]
I. Node⁎	6.6 ± 7.3 [0.0, 39.0]	3.0 ± 4.9 [0.0, 36.0]
T. Node⁎	12.8 ± 6.1 [1.0, 39.0]	11.4 ± 5.4 [1.0, 36.0]
No. Chemo	7.7 ± 0.9 [4.0, 10.0]	7.4 ± 1.3 [1.0, 9.0]

Age (age at diagnosis); NR (lymph node involvement ratio); menarche (menarche age); No. Preg (number of pregnancy); TS (tumor size); Ki67 (Ki67 proliferation marker); I. Node: number of involved auxiliary lymph nodes; T. Node: number of dissected auxiliary lymph nodes; No. Chemo: number of chemotherapy; ND: normally distributed. The sample size was 579 and the recurrence prevalence was 19.3%.

⁎ Statistically significant features (P < 0.05).

**Table 3:** Comparison of clinical and biochemical features (with nominal measurement scale) of included subjects with/without cancer recurrence in percentage.

Variable	With recurrence	Without recurrence
FH	P 18 N: 82	P: 22 N: 78
Multifocal	33 67	27 73
ER	50 50	57 43
PR⁎	50 50	61 39
P53	42 58	34 66
Surgery⁎	MRM: 51 BCS: 14 Mast: 35	56 24 20
Her2	58 42	58 42
Cathepsin	93 7	95 5
HRT⁎	55 43	70 30
XRT	99 1	98 2
Subtypes⁎	LA: 17 LB: 16 HLB: 26 NLH: 31 3N: 10	14 14 26 32 14

FH (family history of cancer); multifocal (having more than one tumor in the breast); ER (estrogen receptor); PR (progesterone receptor); P53 (tumor protein 53); surgery: type of surgery (MRM: modified radical mastectomy, BCS: breast-conserving surgery, Mast: mastectomy); Her2 (epidermal growth factor receptor-2); Cathepsin (Cathepsin-D); HRT (hormone therapy); XRT (radiotherapy); subtypes: cancer molecular subtypes (LA: luminal A, LB: luminal B; HLB: HER2-positive luminal B, NLH: non-luminal Her2, 3N: triple negative). The characteristics were shown as positive and negative percentages (i.e. relative frequent table) for binary variables, respectively and the mode was underlined. The sample size was 579 and the recurrence prevalence was 19.3%.

⁎ Statistically significant features (P < 0.05).

**Table 4:** Comparison of the tumor “histological grade” feature (with ordinal measurement scales) of included subjects with/without cancer recurrence.

Grade categories	With recurrence	Without recurrence
1	22	10
2	44	59
3	33	29
4	1	2

The characteristics were shown as percentages (i.e. relative frequent table), and the mode (the most frequent item) was underlined in each category (recurrent or non-recurrent patient groups). Tumor grade was not statistically significant in subjects with/without recurrence. The sample size was 579 and the recurrence prevalence was 19.3%.

**Table 5:** The holdout performance estimate (%) of the selected classifiers.

Method	Se %	Sp %	Acc %	Pr %	F-score %	Alpha	Beta	AUC	MCC	DOR	DP	Kappa
HPBCR	81	98	90	97	89	0.02	0.19	0.90	0.81	208.9	1.28	0.83
SVM	67	88	78	84	75	0.12	0.33	0.77	0.57	14.9	0.65	0.73
Decision tree	75	78	77	79	77	0.22	0.25	0.76	0.58	10.6	0.57	0.72
MLP	69	81	76	79	73	0.19	0.31	0.75	0.52	10.63	0.57	0.71

Se: sensitivity, Sp: specificity, Acc: accuracy, Pr: precision, AUC: area under the curve, MCC: Matthews correlation coefficient, DOR: diagnostics odds ratio; DP: discriminant power; SVM: supported vector machines; MLP: multilayer perceptron artificial neural network. Seventy percent of the data was used for training and the rest for validation.

**Table 6:** The performance of the classifiers in percent based on 4-fold cross-validation (mean ± SD) [min, max].

Method	Sensitivity	Specificity	Accuracy	Precision
Proposed method	80.0 ± 0.3 [77.0, 80.5]	96.1 ± 1.0 [93.0, 95.0]	89.2 ± 0.6 [85.0, 89.0]	96.5 ± 1.0 [95.0, 98.2]
SVM	73.0 ± 1.0 [66.0, 74.0]	85.3 ± 1.2 [80.0, 89.0]	77.6 ± 0.4 [76.1, 78.9]	82.1 ± 0.3 [80.0, 84.8]
Decision tree	74.0 ± 0.9 [71.8, 76.0]	77.0 ± 0.8 [75.1, 78.5]	77.1 ± 0.2 [76.6, 77.5]	78.1 ± 0.6 [77.3, 80.2]
MLP	67.5 ± 0.2 [66.2, 67.9]	84.3 ± 1.0 [80.8, 85.0]	76.0 ± .0.3 [75.0, 76.8]	84.2 ± 1.8 [79.9, 86.0]

SVM: supported vector machines; MLP: multilayer perceptron artificial neural network.

**Table 7:** The overall confusion matrix of HPBCR on the test set.

	Total population	Condition (as determined by “gold standard”)	
		Condition positive	Condition negative
Test outcome	Test outcome positive	True positive 51	False positive (Type I error) 2
	Test outcome negative	False negative (Type II error) 11	True negative 110

Gold standard: recurrent cancer; test outcome: the decision of the proposed diagnosis system (HPBCR). The classifier was trained on the training set (70% of the samples) and its performance was shown on the test set.

**Table 8:** The holdout performance estimate (%) of the prognosis system with modifications.

Scenario	Se %	Sp %	Acc %	Pr %	F-score %	Alpha	Beta	AUC	MCC	DOR	DP	Kappa
Baseline	81	98	90	97	89	0.02	0.19	0.90	0.81	208.9	1.28	0.83
1	78	81	78	93	86	0.18	0.21	0.79	0.51	15.1	0.65	0.78
2	81	98	90	96	89	0.02	0.19	0.90	0.81	208.9	1.28	0.83
3	80	96	88	97	87	0.04	0.2	0.88	0.79	116.9	1.14	0.81

Baseline: original developed HPBCR; Scenario 1: excluding PSO from the algorithm, 2: using SVM classifier instead of BDT, 3: using MLP classifier instead of BDT, Se: sensitivity, Sp: specificity, Acc: accuracy, Pr: precision, AUC: area under the curve, MCC: Matthews correlation coefficient, DOR: diagnostics odds ratio; DP: discriminant power; SVM: supported vector machines; MLP: multilayer perceptron artificial neural network. Seventy percent of the data was used for training and the rest for validation.
