# Supplementary material for: A Hybrid Computer-aided-diagnosis System for Prediction of Breast Cancer Recurrence (HPBCR) Using Optimized Ensemble Learning
Source: Comput Struct Biotechnol J. 2016 Dec 6;15:75–85. doi: 10.1016/j.csbj.2016.11.004 (PMC5173316; doi:10.1016/j.csbj.2016.11.004)
Supplement: Supplementary material S1 — Publications relevant to cancer recurrence prediction. [file mmc1.docx]

**Supplementary Material (Table S1):** Publications relevant to cancer recurrence prediction^[[1]](#footnote-1)^

| Validation Method | Average  Accuracy (%) | No of Patients | Method | Publication |
| --- | --- | --- | --- | --- |
| Hold-out (80%) | 91 | 97 | Mixture of rough set and SVM | [1] |
| Hold-out (70%) | 91 | 679 | SVM | [2] |
| 10-fold cross validation | 95 | 547 | SVM | [3] |

SVM: Support Vector Machine

**Referencies:**

[1] Zeng T LJ. Mixture classification model based on clinical markers for breast cancer prognosis. Artificial Intelligence in Medicine. 2010;48: 129-37

[2] Kim W, Kim KS, Lee JE, Noh D-Y, Kim S-W, Jung YS, et al. Development of novel breast cancer recurrence prediction model using support vector machine. Journal of breast cancer 2012;15: 230-8

[3] Ahmad L, Eshlaghy A, Poorebrahimi A, Ebrahimi M, Razavi A. Using three machine learning techniques for predicting breast cancer recurrence. Journal of Health & Medical Informatics 2013;4: 124

1. The reader is invited to study the following paper for comprehensive review on the diagnosis, and prognosis (recurrence and survival) :

   Kourou K, Exarchos TP, Exarchos KP, Karamouzis MV, Fotiadis DI. Machine learning applications in cancer prognosis and prediction. Computational and structural biotechnology journal 2015;13: 8-17 [↑](#footnote-ref-1)
