# Supplementary material for: A Hybrid Computer-aided-diagnosis System for Prediction of Breast Cancer Recurrence (HPBCR) Using Optimized Ensemble Learning
Source: Comput Struct Biotechnol J. 2016 Dec 6;15:75–85. doi: 10.1016/j.csbj.2016.11.004 (PMC5173316; doi:10.1016/j.csbj.2016.11.004)
Supplement: Supplementary material S2 — The input features (risk factor) used in the proposed breast cancer recurrence prediction system along with their definition and references. [file mmc2.docx]

**Supplementary Material (Table S2):** The input features (risk factor) used in the proposed breast cancer recurrence prediction system along with their definition and references

| **Measurement Scale** | **Name** | **description** | **references** |
| --- | --- | --- | --- |
| Interval | Age | age at diagnosis of breast cancer | [1] |
|  | NR (Node Ratio) | defined as ratio of involved to dissected lymph nodes, strong prognostic predictor of node-positive patients | [2] |
|  | Menarche | the first [menstrual cycle](https://en.wikipedia.org/wiki/Menstrual_cycle) age | [3] |
|  | No. Preg (Number of pregnancy) | the number of pregnancy | [4] |
|  | T.S (Tumor size) | primary tumor size- smaller tumors, have higher chances for long-term survival | [5] |
|  | Ki67 (%) | Cellular marker for proliferation The fraction of Ki-67-positive tumor cells (the Ki-67 labeling index) is often correlated with the clinical course of cancer.  It is the percentage of tumor cells that are positive for Ki-67. More positive cells tend to more quickly dividing and forming new cells. | [6] |
|  | I. Node (Involved node) | The number of involved axillary lymph nodes | [5] |
|  | T.Node (Dissected node) | The number of dissected axillary lymph nodes | [5] |
|  | No. Chemo (Number of chemo therapies) | The number of chemo therapies | [7] |
| Nominal  (non-binary) | Surgery | type of surgery (MRM: Modified Radical Mastectomy(the entire breast is removed, including the skin, areola, nipple, and most axillary lymph nodes), BCS: Breast-Conserving Surgery (only the part of the breast containing the cancer is removed), Mast: Mastectomy (similar to MRM but the surgeon does not perform axillary lymph node dissection )) | [8] |
|  | Cancer subtypes | Four molecular cancer subtypes: luminal A (ER and/or PR-positive/HER2 negative/low Ki-67^[[1]](#footnote-1)^), luminal B (ER- and/or PR-positive/HER2-negative/high Ki-67), HER2-positive luminal B (ER- and/or PR-positive/HER2 positive/any Ki-67), non-luminal HER2-positive (ER and PR negative/HER2 positive) and triple negative (ER and PR negative/HER2-negative). | [9] |
| Nominal  (binary) | FH (Family history) | family history of cancer (negative/positive) | [10] |
|  | Multifocal | Having more than one distinct tumor within the same quadrant^[[2]](#footnote-2)^ of the breast (negative/positive) | [11] |
|  | ER (Estrogen receptor) | Estrogen receptor expression; cancer cells, like normal breast cells, may receive signals from estrogen that could promote their growth (negative (also known as absent)/positive) | [12] |
|  | PR (Progesterone receptor) | Progesterone receptor expression; cancer cells may receive signals from progesterone that could promote their growth (negative (also known as absent)/positive) | [12] |
|  | p53 (Protein 53)^[[3]](#footnote-3)^ | Phosphoprotein p53 , its mutation is associated with more aggressive disease and worse overall survival (negative/positive) | [13] |
|  | Her2^[[4]](#footnote-4)^ | Epidermal growth factor receptor-2 gene, also called the ERBB2 (Erb-B2 receptor tyrosine kinase 2; Breast cancers with HER2 protein overexpression are called HER2-positive. HER2-positive breast cancers tend to grow faster; (negative/positive (also known as overexpressed)) | [12] |
|  | Cathepsin^[[5]](#footnote-5)^ | Cathepsin-D protein status; Mutations in this gene might be involved the pathogenesis of breast cancer (negative/positive) | [14] |
|  | HRT (hormone therapy) | Using hormone therapy (negative/positive) | [15] |
|  | XRT (radio therapy) | Using radio therapy (negative/positive) | [8] |
| Ordinal | grade | The histological grade of tumor (1 to 4); G1: well differentiated (low grade), G2: Moderately differentiated (intermediate grade), G3: Poorly differentiated (high grade), G4: Undifferentiated (high grade); Tumors with lower grades tend to grow and spread slowly. | [16] |

**References:**

1. Hart, D.W., *Introduction to power electronics*. Vol. 11. 1997: Prentice Hall New Jersey.

2. Collins, L.C., et al., *Pathologic features and molecular phenotype by patient age in a large cohort of young women with breast cancer.* Breast Cancer Research and Treatment, 2012. **131**(3): p. 1061-6.

3. Sotiriou, C., et al., *Breast cancer classification and prognosis based on gene expression profiles from a population-based study.* Proc Natl Acad Sci U S A, 2003. **100**(18): p. 10393-8.

4. Marateb, H.R., et al., *A hybrid intelligent system for diagnosing microalbuminuria in type 2 diabetes patients without having to measure urinary albumin.* Comput Biol Med, 2014. **45**: p. 34-42.

5. Chawla, N.V., *Data Mining for Imbalanced Datasets: An Overview*, in *Data Mining and Knowledge Discovery Handbook*, O. Maimon and L. Rokach, Editors. 2005, Springer US: Boston, MA. p. 853-867.

6. Mosteller, F., *A k-Sample Slippage Test for an Extreme Population.* The Annals of Mathematical Statistics, 1948. **19**(1): p. 58-65.

7. Rubin, A., *Statistics for evidence-based practice and evaluation*. 3rd ed. 2013, Belmont, CA: Brooks/Cole, Cengage Learning. xvi, 347 p.

8. Cowles, M. and C. Davis, *On the origins of the. 05 level of statistical significance.* American Psychologist, 1982. **37**(5): p. 553.

9. Shim, H.J., et al., *Breast cancer recurrence according to molecular subtype.* Asian Pac J Cancer Prev, 2014. **15**(14): p. 5539-44.

10. Marateb, H.R., et al., *Manipulating measurement scales in medical statistical analysis and data mining: A review of methodologies.* J Res Med Sci, 2014. **19**(1): p. 47-56.

11. Lin, R. and P. Tripuraneni, *Radiation Therapy in Early-Stage Invasive Breast Cancer.* Indian journal of surgical oncology, 2011. **2**(2): p. 101-111.

12. Banerjee, A., et al., *Hypothesis testing, type I and type II errors.* Industrial Psychiatry Journal, 2009. **18**(2): p. 127-131.

13. Smith, P.W., *Transient electronics: pulsed circuit technology*. 2011: John Wiley & Sons.

14. Stigler, S., *Fisher and the 5% level.* CHANCE, 2008. **21**(4): p. 12-12.

15. Ellis, P.D., *The essential guide to effect sizes : statistical power, meta-analysis, and the interpretation of research results*. 2010, Cambridge ; New York: Cambridge University Press. xvii, 173 p.

16. Guilherme, H.M.O., et al., *Mayo Clinic Medical Manual and Mayo Clinic Internal Medicine Review*. 7th ed. 2007, Rochester, MN, USA: CRC Press

1. The cutoff value of 14% was used to define ‘low’ and ‘high’ [↑](#footnote-ref-1)
2. <https://training.seer.cancer.gov/breast/anatomy/quadrants.html> [↑](#footnote-ref-2)
3. <https://www.ncbi.nlm.nih.gov/gene/7157> [↑](#footnote-ref-3)
4. <https://www.ncbi.nlm.nih.gov/gene/2064> [↑](#footnote-ref-4)
5. <https://www.ncbi.nlm.nih.gov/gene/1509> [↑](#footnote-ref-5)
