# Supplementary material for: A Hybrid Computer-aided-diagnosis System for Prediction of Breast Cancer Recurrence (HPBCR) Using Optimized Ensemble Learning
Source: Comput Struct Biotechnol J. 2016 Dec 6;15:75–85. doi: 10.1016/j.csbj.2016.11.004 (PMC5173316; doi:10.1016/j.csbj.2016.11.004)
Supplement: Supplementary material S3 — The pseudo code of the proposed algorithm. [file mmc3.docx]

**Supplementary Material (Table S3):** The pseudo code of the proposed algorithm

**Input:** the mixed-type data^[[1]](#footnote-1)^ for a two-class prediction problem

**Output:** the selected features along with their estimated weights, and the Bagged Decision Tree (BDT) structure and the recurrence/no recurrence output for each sample in the test set

Split the dataset into the training and test sets.

Set ‘iter’ variable to zero.

MAX_ITER=100;

For the training dataset:

1. Using Statistical Feature Selection (SFS) to identify discriminative features
   - For features with nominal measurement scale, run chi-square test^[[2]](#footnote-2)^ and select those that are statistically significant (P<0.05)
   - For features with ordinal measurement scale, run Mann–Whitney U test^[[3]](#footnote-3)^ and select those that are statistically significant (P<0.05)
   - For features with interval measurement scale, check whether they are normally distributed using the Kolmogorov-Smirnov (KS) test^[[4]](#footnote-4)^
     - For normally-distributed features, run independent-sample t-test^[[5]](#footnote-5)^ and select those that are statistically significant (P<0.05)
     - For features not normally-distributed, run Mann–Whitney U test and select those that are statistically significant (P<0.05)
2. Using embedded FS on the remained features based on Particle Swarm Optimization (PSO) to further select categorical features and to tune the weight of features with interval measurement scale
   - Do random assignment of weights of features between 0, and 1 for a number of particles (i.e. possible solutions; 20 in our algorithm)
   - B.1) iter=iter+1;
   - For each particle, calculate the fitness value on the training set data
     - Include categorical features whose weight is greater than or equal to 0.5 (set F1)
     - Multiply interval features by their weights (set F2)
     - Design the bagged Decision Tree (BGD) classifier using {F1,F2} set (C4.5 DT algorithm is used and the number of trees was set to 10 in our algorithm)
     - Calculate Precision (Pr) and Sensitivity (Se) and then F_1_-score as the harmonic mean of Pr and Se as the fitness value in the designed classifier on the training set
   - Update the personal best (pbest) i.e. the best fitness value a particle had so far
     - For each particle IF the new fitness is higher than its previous pbest, THEN update pbest and save related BDT structure and feature weights.
   - Find the maximum pbest of the entire particles (named as gbest)^[[6]](#footnote-6)^ and save related BDT structure and feature weights.
   - IF gbest does not significantly change during 10 consecutive iterations or the number of iterations (iter) is more than MAX_ITER then STOP. GOTO (C)
   - For each particle, calculate the velocity using the EQ (2) in the manuscript.
   - Update the feature weights using the EQ (3) in the manuscript.
   - GOTO (B.1)
3. Report the BDT structure and feature weights. Keep categorical features whose weights are greater than or equal to 0.5 and also interval features whose weight is greater than 0.1 (set F3)

For the test dataset:

Use features from set F3.

Multiply selected interval features by the estimated weights after training.

Run BDT obtained from training procedure with these new features.

The output of the classifier for each subject is either recurrence or no recurrence.

1. Mixed-type data includes features whose measurement scale are different (i.e. nominal, ordinal or interval). Categorical data includes nominal and ordinal data. [↑](#footnote-ref-1)
2. The Chi-square test is use to identify whether the exposure (a nominal feature) and outcome (cancer recurrence) are significantly dependent (P<0.05). [↑](#footnote-ref-2)
3. The Mann–Whitney U test (also known as the Mann–Whitney–Wilcoxon, Wilcoxon rank-sum test, or Wilcoxon–Mann–Whitney test) is used to identify whether the mean rank of an ordinal feature in two (cancer recurrence/no-recurrence) groups is significantly different (P<0.05). [↑](#footnote-ref-3)
4. The Kolmogorov-Smirnov (KS) test is usually used to check the normality of (large-sample) data with interval measurement scale. The null hypothesis in this test is that the data is normality distributed. Thus, is met when P>0.05. [↑](#footnote-ref-4)
5. The independent-sample t-test is used to identify whether the average of a normally-distributed feature in two (cancer recurrence/no-recurrence) groups is significantly different (P<0.05). [↑](#footnote-ref-5)
6. The star topology was used in our study. [↑](#footnote-ref-6)
