# Supplementary material for: A Hybrid Computer-aided-diagnosis System for Prediction of Breast Cancer Recurrence (HPBCR) Using Optimized Ensemble Learning
Source: Comput Struct Biotechnol J. 2016 Dec 6;15:75–85. doi: 10.1016/j.csbj.2016.11.004 (PMC5173316; doi:10.1016/j.csbj.2016.11.004)
Supplement: Supplementary material S4 — Statistical analysis of the validation measures. [file mmc4.docx]

**Supplementary material S4:** Statistical analysis of the validation measures

Suppose that we want to assess the performance of a computer-aided-diagnosis (CAD) system. This is accomplished by comparing its results with that of gold standard (e.g. pathology results) using traditional signal detection theory [1].

The confusion matrix could be constructed for such a validation. Let D be the event that the tested person has the disease (e.g. recurrence as confirmed by the oncologist) and E the event that his test result is positive (e.g. the CAD system output is recurrence). It is easily shown that there is a relationship between resulting probabilities [2] and signal detection theory parameters.

**Table A3.1:** The overall confusion matrix of a CAD system

|  | | **Condition (as determined by "Gold standard")** | |
| --- | --- | --- | --- |
|  | Total population | Condition positive  Event (D) | Condition negative  Event (D^c^) |
| **Test outcome** | Test outcome positive  Event (E) | **True Positive**  TP | **False Positive** (Type I error)  FP |
|  | Test outcome negative  Event (E^c^) | **False Negative** (Type II error)  FN | **True Negative**  TN |

Total sample size (S) is equal to the sum of TP, TN, FP and FN; the complement of an event A is shown as A^c^.

In fact, the following statements could be obtained using the tree diagram:

$\left\{\begin{matrix} TP=P\left(ED\right)\times S=P(E\backslash D)\times P(D)\times S \\ TN=P\left(E^{c}D^{c}\right)\times S=P(E^{c}\backslash D^{c})\times P(D^{c})\times S \\ FP=P\left(ED^{c}\right)\times S=P(E\backslash D^{c})\times P(D^{c})\times S \\ TP=P\left(E^{c}D\right)\times S=P(E^{c}\backslash D)\times P(D)\times S \end{matrix}\right.$ (EQ. 1)

where *P(D)* is the prevalence of the disease (P) and *P(D^c^)* is thus one minus prevalence (1-P). Meanwhile, the following equalities hold between performance indices and conditional probabilities:

$\left\{\begin{matrix} Se=P\left(E\backslash D\right) \\ Sp=P\left(E^{c}\backslash D^{c}\right) \\ \alpha=1-Sp=P\left(E\backslash D^{c}\right) \\ \beta=1-Se=P\left(E^{c}\backslash D\right) \end{matrix}\right.$ (EQ. 2)

where Se, Sp, $\alpha$ and $\beta$ are Sensitivity, Specificity, Type I and II errors, respectively. In fact not only P(E\D) (i.e. sensitivity) is important but also the conditional posterior probability P(D\E) is important. It is the probability a person has the disease given that his test result is positive. Using Bayes’ Formula, it is possible to calculate this posterior probability.

$P(D\backslash E)=\frac{P(E\backslash D)\times P(D)}{P(E\backslash D)\times P(D)+P(E\backslash D^{c})\times P(D^{c})}$ (EQ. 3)

Combining EQ 2 and 3, the following statement is obtained:

$P(D\backslash E)=\frac{Se\times P}{Se\times P+\left(1-Sp\right)\times\left(1-P\right)}$ (EQ. 4)

The posterior probability is highly dependent on the prevalence of the disease. For example, assume that a diagnosis test has the sensitivity and specificity of 80%, and 95% receptively and the prevalence of the disease is 10%. The posterior probability is 64%. In extreme case where the disease is rare (e.g. P=0.5%), even if the sensitivity and specificity are both 99%, the posterior probability decreases down to 33%. Thus, using only the condition of Se and Sp greater than 80% and 95%, respectively is not enough.

Interestingly, such a posterior probability is nothing but the criterion Precision (=PPV) defined as TP/(TP+FP). The acceptable range for PPV is, however, dependent on the disease prevalence. Setting Se and Sp to 80% and 95%, with the prevalence of 20%, PPV is 80%. However, it was shown in the literature that a good CAD system must have the precision of not less than 95% [3]. Thus, Se and Sp must be higher to satisfy this condition.

The diagnostic Odds ratio (DOR), unlike Se and Sp, is not dependent on the disease prevalence and was shown to be a single global indicator of CAD performance [4, 5].

**References:**

1. McNicol, D., *A primer of signal detection theory*. 2005: Psychology Press.

2. Ross, S.M., *Introduction to probability and statistics for engineers and scientists*. 2014: Academic Press.

3. Colquhoun, D., *An investigation of the false discovery rate and the misinterpretation of p-values.* Open Science, 2014. **1**(3): p. 140216.

4. Glas, A.S., et al., *The diagnostic odds ratio: a single indicator of test performance.* Journal of Clinical Epidemiology, 2003. **56**(11): p. 1129-1135.

5. Šimundić, A.-M., *Measures of diagnostic accuracy: basic definitions.* Med Biol Sci, 2008. **22**(4): p. 61-5.
